# Влияние компонентов ренин-ангиотензиновой системы, полиморфизма rs2106809 гена <i>ACE2</i> и терапии блокаторами РАС на тяжесть течения COVID-19

**DOI:** 10.14341/probl13274

**Published:** 2023-08-30

**Authors:** З. Т. Зураева, О. К. Викулова, Н. М. Малышева, Л. В. Никанкина, Н. В. Зайцева, О. Ю. Сухарева, М. Ш. Шамхалова, М. В. Шестакова, Н. Г. Мокрышева

**Affiliations:** Национальный медицинский исследовательский центр эндокринологии; Национальный медицинский исследовательский центр эндокринологии; Национальный медицинский исследовательский центр эндокринологии; Национальный медицинский исследовательский центр эндокринологии; Национальный медицинский исследовательский центр эндокринологии; Национальный медицинский исследовательский центр эндокринологии; Национальный медицинский исследовательский центр эндокринологии; Национальный медицинский исследовательский центр эндокринологии; Национальный медицинский исследовательский центр эндокринологии

**Keywords:** COVID-19, SARS-CoV-2, ренин-ангиотензиновая система, ангиотензинпревращающий фермент 2, ангиотензин II, ADAM17, ингибиторы АПФ, блокаторы рецепторов ангиотензина, полиморфизм rs2106809

## Abstract

ОБОСНОВАНИЕ. Ангиотензинпревращающий фермент 2 (АПФ2) является одним из ключевых компонентов ренин-ангиотензиновой системы (РАС), обеспечивающим контррегуляцию ее эффектов, и одновременно рецептором для проникновения в клетку коронавируса SARS-CoV-2, вызывающего развитие COVID-19. Предполагается, что факторы, регулирующие баланс основных компонентов РАС, в том числе полиморфизм гена ACE2, терапия блокаторами РАС (ингибиторами АПФ (иАПФ) и блокаторами рецепторов ангиотензина (БРА)), могут оказывать влияние на тяжесть течения COVID-19.ЦЕЛЬ. Цель исследования — изучить влияние уровня циркулирующих компонентов РАС, аллелей и генотипов полиморфизма rs2106809 гена ACE2 и предшествовавшей развитию COVID-19 терапии препаратами иАПФ/БРА на тяжесть течения инфекции.МАТЕРИАЛЫ И МЕТОДЫ. В исследование включены пациенты с COVID-19, госпитализированные в НМИЦ эндокринологии (n=173), которые были распределены на группы среднетяжелого и тяжелого течения. Определение компонентов РАС осуществляли методом иммуноферментного анализа, идентификацию полиморфизма — полимеразной цепной реакцией в режиме реального времени.Статистический анализ проводили с помощью методов непараметрической статистики, различия в распределении частот генотипов оценивали посредством точного критерия Фишера χ2.РЕЗУЛЬТАТЫ. Группы среднетяжелого и тяжелого течения COVID-19 достоверно различались по возрасту, уровням глюкозы крови, воспалительных маркеров: лейкоцитов, нейтрофилов, IL-6, D-димера, C-реактивного белка, ферритина и активности печеночных ферментов, которые коррелировали с тяжестью течения заболевания. При сравнении пациентов по уровням АПФ, АПФ2, ангиотензина II, ADAM17 не было выявлено статистически значимых различий исследованных компонентов РАС между группами среднетяжелого и тяжелого течения (р=0,544; р=0,054; р=0,836; р=1,0 соответственно), в том числе с учетом распределения по полу (у мужчин: р=0,695; р=0,726; р=0,824; р=0,512; у женщин: р=0,873; р=0,196; р=0,150; р=0,937).Анализ распределения генотипов АА, AG, GG полиморфизма rs2106809 гена ACE2 также не выявил достоверных различий между пациентами с различной тяжестью течения: χ2=1,35, р=0,071 у мужчин, χ2=5,28, р=0,244 у женщин. Значимых различий в использовании блокаторов РАС в группах с разной тяжестью течения выявлено не было: χ2=0,208, р=0,648 для иАПФ, χ2=1,15, р=0,283 для БРА.ЗАКЛЮЧЕНИЕ. В нашем исследовании гипотеза о влиянии активации компонентов РАС (АПФ, АПФ2, ангиотензина II, ADAM17) и полиморфизма гена ACE2 на тяжесть течения COVID-19 не подтвердилась. Степень тяжести клинических проявлений COVID-19 значимо коррелировала с уровнем стандартных воспалительных маркеров, что указывает на общие принципы течения инфекции как системного воспаления, вне зависимости от генетического и функционального статуса РАС.

## Abstract (en)

BACKGROUND: Angiotensin-converting enzyme 2 (ACE2) is a key component of the renin-angiotensin system (RAS), providing counter-regulation of its effects and, simultaneously, a receptor for the SARS-CoV-2 entering. It is suggested that factors regulating the balance of the major components of RAS, including ACE2 gene polymorphism, therapy with RAS blockers (ACE inhibitors and angiotensin receptor blockers) — may affect the severity of COVID-19.AIM: The aim of the study was to investigate the effect of RAS components, the relationship of ACE2 gene polymorphism rs2106809 and ACEi/ARBs therapy with the COVID-19 severity.MATERIALS AND METHODS: The study included patients with COVID-19 hospitalized in Endocrinology research centre (n = 173), who were divided into groups of moderate and severe course. Determination of RAS components was performed by ELISA, identification of polymorphism by PCR. Statistical analysis was performed using nonparametric statistical methods; differences in the distribution of genotype frequencies were assessed using Fisher’s exact test χ2.RESULTS: The groups differed significantly in age, blood glucose levels, and inflammatory markers: leukocytes, neutrophils, IL-6, D-dimer, C-reactive protein, ferritin and liver enzymes, which correlated with the severity of the disease. When comparing patients in terms of ACE, ACE2, angiotensin II, ADAM17 there were no statistically significant differences between the groups (p=0.544, p=0.054, p=0.836, p=1.0, respectively), including the distribution by gender (in men: p=0.695, p=0.726, p=0.824, p=0.512; in women: p=0.873, p=0.196, p=0.150, p=0.937). Analysis of the distribution of AA, AG, and GG genotypes of the rs2106809 polymorphism of the ACE2 gene also revealed no differences between patients: χ2 1.35, p=0.071 in men, χ2 5.28, p=0.244 in women. There were no significant differences in the use of RAS blockers between groups with different course severity: χ2 0.208, p=0.648 for ACEi, χ2 1.15, p=0.283 for ARBs.CONCLUSION: In our study, the influence of activation of RAS components (ACE, ACE2, AT II, ADAM17) and ACE2 gene polymorphism on the severity of COVID-19 course was not confirmed. The severity of COVID-19 course correlated with the level of standard inflammatory markers, indicating the general principles of the infection as a systemic inflammation, regardless of the genetic and functional status of the RAS.

## ОБОСНОВАНИЕ

Глобальный рост заболеваемости новой коронавирусной инфекцией (COVID-19) и тяжелые социально-экономические последствия пандемии определили чрезвычайную важность выявления факторов риска тяжелого течения и смерти, а также разработки эффективной терапии. Ангиотензинпревращающий фермент 2 типа (АПФ2) является функциональным рецептором SARS-Cov-2 для проникновения вируса в клетки, что обусловило предпосылки для изучения влияния основных компонентов ренин-ангиотензиновой системы (РАС) на предрасположенность к более тяжелому течению заболевания и развитию неблагоприятных клинических исходов [1]. Классическая концепция РАС основана на балансе взаимодействия локальной регуляторной оси АПФ-ангиотензин II(АТII)/рецепторы к ангиотензину и контррегуляторной оси АПФ2-ангиотензин (1–7) (АТ (1–7))/Мас-рецепторы [2]. Предполагается, что после связывания вируса с рецептором АПФ2, последний оказывает влияние на количество и активность АТII, что приводит к усилению провоспалительного эффекта через AT1–рецепторный механизм. Помимо этого, АТII является мощным вазопрессором, стимулятором фиброза и синтеза альдостерона. Система АТII-рAT1 активирует дезинтегрин и металлопротеиназу ADAM17, что приводит к внутриклеточной продукции эпидермального фактора роста, лигандов и фактора некроза опухолей α (ФНОα), которые, в свою очередь, стимулируют транскрипцию сигнального пути ядерного фактора NF-κB — ключевого компонента в провоспалительном цитокиновом ответе. Механизм является самоподдерживающимся, поскольку NF-κB индуцирует экспрессию гена ангиотензиногена, усиливая воспалительный ответ АТII. АПФ2, осуществляя гидролиз АТII до вазодепрессорного пептида АТ (1–7), является ключевым ферментом защитной оси РАС. Результирующим эффектом стимуляции регуляторной оси АПФ2-АТ (1–7) является активация протективного антикоагуляционного, противовоспалительного, антипролиферативного, антифибротического действия, замедление апоптоза эпителия альвеолярных клеток и процессов оксидативного стресса, противоположных негативным эффектам АТII [3][4].

Потенциальное влияние на тяжесть и клинические исходы коронавирусной инфекции могут оказывать индивидуальные различия в уровне циркулирующих компонентов РАС и экспрессии рецепторов, опосредованные возрастными, гендерными особенностями, факторами окружающей среды, а также полиморфизмом генов ферментной системы и приемом лекарственных препаратов, модулирующих активность РАС.

Известно, что экспрессия АПФ2 в легких значительно снижается с возрастом и в более значительной степени у мужчин, чем у женщин, что отчасти может объяснять более высокий риск неблагоприятных исходов у пожилых пациентов и мужчин [5]. До 67% фенотипических вариаций циркулирующего АПФ2 может быть обусловлено генетическими факторами [6]. Имеющиеся данные указывают на важную функциональную роль полиморфных вариантов гена ACE2 в развитии кардиоваскулярных и легочных заболеваний, что, в свою очередь, может также определять особенности клинического течения COVID-19 [7]. Ген ACE2 обладает высокой степенью генетических полиморфизмов, включая точечные генетические варианты (однонуклеотидные полиморфизмы, SNP), транскрипционные вариации, посттранскрипционные модификации белка, среди которых SNP являются наиболее активно изучаемыми. SNP, определяющие различия в экспрессии генов и функции белков, способны влиять на появление тех или иных фенотипических особенностей и вносить вклад в индивидуальную предрасположенность к ряду заболеваний и особенностям их течения. Отмечается наличие гендерных различий во взаимосвязи между SNP гена ACE2 и некоторыми заболеваниями. Предполагается, что среди различных полиморфизмов гена ACE2 полиморфизм rs2106809 является одним из наиболее перспективных для изучения патогенетических аспектов вовлеченности РАС в развитие различных осложнений, в том числе связанных с COVID-19. Так, по данным одного из исследований по оценке различных вариантов гена ACE2, установлена связь данного полиморфизма с уровнем экспрессии гена и концентрации АПФ в крови, что определяет выраженность модулируемых АТII нарушений. Предполагается, что именно SNP rs2106809 может создавать интронно-экзонный усиливающий сайт, оказывающий влияние на эффективность сплайсинга АПФ2 [8].

Особую настороженность вызвали сообщения о влиянии фармакологического ингибирования РАС на повышение экспрессии АПФ2 и, соответственно, усиление вирулентности SARS-CoV-2 в легочной системе из-за связывания S-белка вируса с АПФ2 и его активного проникновения в клетки [9]. До настоящего времени окончательно не решен вопрос о влиянии терапии блокаторами РАС (ингибиторами АПФ (иАПФ) и блокаторами рецептора ангиотензина II (БРА)) на течение и риск развития неблагоприятных исходов при COVID-19.

РАС является не только ключевым модулятором проникновения вируса в клетки, но также триггером каскада патологических реакций, ведущих к усилению провоспалительного ответа, опосредующего негативные эффекты инфекции, что и определило цели и задачи нашего исследования.

## ЦЕЛЬ ИССЛЕДОВАНИЯ

Оценить влияние клинико-демографических, метаболических и генетических факторов активности РАС, терапии ингибиторами РАС (иАПФ/БРА) на тяжесть течения и клинические исходы вирусной инфекции, вызванной SARS-CoV-2.

## МАТЕРИАЛЫ И МЕТОДЫ

## Место и время проведения исследования

Исследование выполнено в период функционирования на базе ФГБУ «НМИЦ эндокринологии» (г. Москва) инфекционного стационара для оказания медицинской помощи пациентам с эндокринными заболеваниями, которые были госпитализированы с новой коронавирусной инфекцией в период с 05 мая по 01 июля 2020 г.

## Изучаемые популяции (одна или несколько)

В исследование были включены пациенты с COVID-19, находившиеся на лечении в ФГБУ «НМИЦ эндокринологии». Диагностика коронавирусной инфекции осуществлялась на основании выявления РНК SARS-CoV-2 с помощью метода амплификации нуклеиновых кислот (ПЦР). Период проведения исследования соответствовал «первой волне» COVID-19, в период которой превалировал бета-штамм инфекции, но в рамках исследования верификация штамма SARS-CoV-2 не проводилась.

Стратификацию пациентов на группы осуществляли в соответствии со степенью тяжести клинического течения: среднее и тяжелое.

Критерии среднетяжелого течения:

Критерии тяжелого течения заболевания:

Специфическое этиотропное лечение COVID-19 осуществлялось в соответствии со стандартным протоколом терапии, согласно действующим на данный период рекомендациям Минздрава России [10].

## Способ формирования выборки из изучаемой популяции (или нескольких выборок из нескольких изучаемых популяций)

Выборка формировалась сплошным методом.

## Дизайн исследования

Ретроспективное одномоментное одноцентровое исследование.

## Методы

Анализируемые сведения были получены из медицинских карт участников исследования. Оценивали антропометрические, демографические данные, сведения о наличии сопутствующей патологии (гипертоническая болезнь, сердечная недостаточность, сахарный диабет, дислипидемия, онкология), сопутствующей сахароснижающей терапии (ССТ) и терапии блокаторами РАС, данные лабораторных стандартных и специальных исследований, выполненных в соответствии с протоколом исследования.

Иммуноферментный анализ.

Определение циркулирующих компонентов РАС осуществляли методом иммуноферментного анализа ELISA в соответствии с инструкциями производителей с использованием следующих тест-систем: АПФ, RayBiotech, США (минимальный детектируемый уровень 0,084 нг/мл), ангиотензин II, RayBiotech, США (минимальный детектируемый уровень 0,3 пг/мл), ADAM17, RayBiotech, США (минимальный детектируемый уровень 70 пг/мл), АПФ2, Cloud-Clone, США (минимальный детектируемый уровень 5,5 пг/мл).

Генетический анализ.

Выделение геномной ДНК из крови пациентов осуществлялось с помощью прибора MagNa Pure 2.0 (Roche) c помощью стандартного протокола MagNa Pure LC DNA Isolation Kit 1 (Roche).

Генотипирование rs2106809 с помощью ПЦР с детекцией результатов в режиме реального времени с использованием taqman-проб.

В реакционную смесь добавляли ДНК-пробы (Taqman-пробы), меченные на 5’-конце флуоресцентным красителем и гасителем на 3’-конце, соответствующий буфер, 2,5 мМ хлорид магния, 0,25 мМ dNTP, прямой и обратный праймеры в концентрации 0,25 мкМ, 1х ROX (Kapa Biosystems), HotTaq полимеразу 2,5 ед./мкл (Евроген) и ДНК-матрицу. Количественную ПЦР (кПЦР) проводили в амплификаторе StepOnePlus (Applied BioSystems). Снятие показаний флуоресценции осуществлялось на стадии отжига праймеров.

Секвенирование ДНК по Сэнгеру.

Для проверки результатов генотипирования, полученных с помощью кПЦР, 16 образцов были проверены с помощью секвенирования по Сэнгеру. В реакционную смесь добавляли соответствующий буфер, 2,5 мМ хлорид магния, 0,25 мМ dNTP, прямой и обратный праймеры в концентрации 0,5 мкМ, Taq полимеразу 2,5 ед./мкл (Синтол) и ДНК-матрицу. ПЦР проводили в амплификаторе. Ферментативная очистка ПЦР-фрагментов осуществлялась с помощью щелочной фосфатазы FastAP 1 ед./мкл (Thermo Scientific) и экзонуклеазы 10 ед./мкл (Thermo Scientific). После ферментативной очистки ДНК-фрагменты смешивались с прямым праймером в концентрации 0,5 мкМ и 2 мкл 10 мM Tris / 0,01 мM EDTA (pH 8.0) и инкубировались в течение 5 мин при 98°C, а затем смесь помещалась в лед. После охлаждения смеси на льду к полученной смеси добавляли реактивы для секвенирования согласно протоколу Applied Biosystems 3.1. ПЦР проводили в амплификаторе. К раствору с ПЦР-фрагментами добавляли 1 мкл 3М ацетата натрия рН 5,2 и 100 мкл 96% этанола. Центрифугировали в течение 30 мин при скорости 4500 об./мин., 4°C. Промывали осадок 100 мкл 70% этанола, высушивали при 72 °C и растворяли в 10 мкл Formamide. Затем полученный раствор нагревали 2 мин при 95 °C. После нагревания реакционную смесь запускали в капиллярный секвенатор 3500, Life Technologies.

## Статистический анализ

Статистический анализ проводился в пакете прикладных программ IBM SPSSv23 Statistics (США) и MedCalc, версия 18.2.1. (MedCalc Software, Бельгия).

Проверка соответствия распределения количественных признаков нормальному закону осуществлялась с использованием теста Шапиро–Уилка. Данные с асимметричным распределением анализировались с использованием методов непараметрической статистики. Описательная статистика параметров, приводимых далее в таблицах, представлена как медианы, первый и третий квартили (Me [Q1; Q3]); n — объем анализируемой подгруппы, р — достигнутый уровень статистической значимости. Сравнение двух независимых выборок для количественных параметров осуществлялось с использованием критерия Манна–Уитни (U-тест). Для оценки значимости качественных характеристик выборки использовался анализ таблиц сопряженности с применением критерия хи-квадрат (χ²). Критический уровень статистической значимости при проверке статистических гипотез принимали равным p<0,05. Корреляцию клинических и лабораторных параметров со степенью тяжести коронавирусной инфекции определяли с помощью рангового корреляционного анализа Спирмена. Пошаговый регрессионный анализ был проведен для проверки относительного вклада различных ковариат (независимых переменных) в тяжесть течения коронавирусной инфекции (зависимая переменная). Ковариаты были выбраны на основе однофакторного анализа (р<0,05).

Сравнение генетических полиморфизмов пациентов проводили с использованием критерия χ² у пациентов с различной степенью тяжести инфекции.

## Этическая экспертиза

В исследование были включены истории болезни пациентов, которые в период лечения в инфекционном стационаре ФГБУ «НМИЦ эндокринологии» добровольно в письменной форме выразили согласие на участие в данном исследовании и использование своей медицинской информации в научных целях. Исследование одобрено на заседании локального этического комитета ФГБУ «НМИЦ эндокринологии», протокол № 6 от 30 апреля 2020 г.

## РЕЗУЛЬТАТЫ

В исследование включены данные 173 пациентов (81 женщина, 92 мужчин), из них 144 пациента из группы среднетяжелого течения, 29 пациентов из группы тяжелого течения заболевания. Основные демографические характеристики пациентов и сопутствующие заболевания представлены в таблице 1. Средняя продолжительность госпитализации составила 12±4 дней, в группе среднетяжелого течения — 12±3 дней, в группе тяжелого течения — 13±6 дней. По данным рентгенологического исследования (компьютерная томография, КТ) 1-я степень поражения легких диагностирована у 20 пациентов (11,5%), КТ2 — у 55 пациентов (31,7%), КТ3 — у 84 пациентов (48,5%), КТ4 — у 14 пациентов (8%). Основные клинические симптомы заболевания: кашель, одышка различной степени выраженности, гастроинтестинальные симптомы были отмечены у 29,5, 57 и 11,5 % пациентов соответственно.

**Table:** Таблица 1. Клиническая характеристика пациентов среднетяжелого и тяжелого течения COVID-19 * критерий Манна–Уитни (U-тест).** критерий χ².Примечания: ИМТ — индекс массы тела, ДАД — диастолическое артериальное давление, САД — систолическое артериальное давление, ЧСС — частота сердечных сокращений, ЧДД — частота дыхательных движений, SpO2 — парциальное давление кислорода, ССЗ — сердечно-сосудистые заболевания.

Параметр	Общая (n=173) Ме [ Q1; Q3]	Среднее (n=144) Ме [ Q1; Q3]	Тяжелое (n=29) Ме [ Q1; Q3]	р*
Возраст	59 [ 48; 73]	58 [ 47; 71]	69 [ 54; 79]	0,027
Пол (м/ж)	92/81	79/65	13/16	
Вес, кг	85 [ 73;97]	85 [ 73; 96]	90 [ 73; 105]	0,209
ИМТ, кг/м²	30 [ 26;34]	29 [ 25; 33]	32 [ 26; 37]	0,086
САД, мм рт.ст.	130 [ 120; 147]	130 [ 120; 145]	140 [ 122; 158]	0,099
ДАД, мм рт.ст.	82 [ 76; 90]	83 [ 76; 90]	80 [ 79; 90]	0,997
ЧСС в минуту	90 [ 78; 101]	89 [ 78; 100]	92 [ 81; 103]	0,337
ЧДД в минуту	22 [ 20; 28]	22 [ 20; 26]	28 [ 23; 32]	0,001
SpO2, %	94 [ 91; 96]	94 [ 92; 97]	88 [ 83; 92]	0,001
Сопутствующие заболевания
Артериальная гипертензия	85 (49,1%)	68 (47%)	17 (58%)	0,263**
ССЗ	34 (19,6%)	28 (19%)	6 (20,6%)	0,878**
Сахарный диабет	35 (20%)	25 (17%)	10 (3,4%)	0,037**
Ожирение	72 (41,6 %)	57 (39,5%)	15 (51,7%)	0,227**
Заболевания легких	14 (8%)	14 (9,7%)	0	0,080**
Онкология	4 (2,3%)	2 (1,4%)	2 (6,8%)	0,072**

Группы среднетяжелого и тяжелого течения достоверно различались по возрасту, р=0,027. Не было выявлено статистически значимых различий по полу, индексу массы тела (ИМТ) и основным клиническим параметрам (АД, ЧСС) (табл. 1). В группе тяжелого течения отмечена тенденция к более высоким значениям ИМТ (кг/м²), не достигавшая статистической значимости (р=0,086) (см. табл. 1). Получены достоверные различия в уровне ЧДД (р=0,001) и SpO2 (р=0,001), что определяется степенью тяжести заболевания.

Частота распространенности артериальной гипертензии (р=0,263), сердечно-сосудистых заболеваний (р=0,878), заболеваний легких (р=0,080) и онкологических заболеваний (р=0,072) достоверно не различалась между группами среднетяжелого и тяжелого течения (см. табл. 1). В группе среднетяжелого течения было больше пациентов с сопутствующим сахарным диабетом, чем в группе тяжелого течения (р=0,037), что, по всей видимости, обусловлено значимо бóльшим количеством пациентов в данной группе.

ССТ у пациентов с СД: из общего количества пациентов 35 имели СД 2 типа (20%). Инсулинотерапия в базальном или базис-болюсном режиме проводилась у 11 пациентов (6,3%), пероральная ССТ включала моно- или комбинированную терапию препаратами метформина — у 17 (9,8%), сульфонилмочевины — у 2 (1,1%), ингибиторами дипептидилпептидазы-4 — у 6 (3,4%), ингибиторами натрий-глюкозного котранспортера 2 типа — у 2 пациентов (1,1%).

У пациентов с тяжелым течением заболевания отмечены достоверно более высокие значения глюкозы (р=0,008), различия в уровне гликированного гемоглобина между группами не достигли статистической значимости (р=0,078). Показатели воспалительных маркеров: лейкоцитов (р=0,005), нейтрофилов (р=0,002), АСТ (р=0,002), ЛДГ (р=0,001), IL-6 (0,002), D-димера (р=0,002), С-реактивного белка (р=0,001) и ферритина (р=0,025) достоверно различались между группами (табл. 2).

**Table:** Таблица 2. Сравнение пациентов среднетяжелого и тяжелого течения по лабораторным показателям * критерий Манн-Уитни (U-тест)Примечания: АСТ — аспартатаминотрансфераза, АЛТ — аланинаминотрансфераза, ЛДГ — лактатдегидрогеназа, IL-6 — интерлейкин-6, СКФ — скорость клубочковой фильтрации, СОЭ — скорость оседания эритроцитов, СРБ — С-реактивный белок.

Параметр	Общая (n=173) Ме [ Q1; Q3]	Среднее (n=144) Ме [ Q1; Q3]	Тяжелое (n=29) Ме [ Q1; Q3]	р*
Глюкоза, моль/л	6,3 [ 5,7; 7,2]	6,1 [ 5,7; 7,0]	7,2 [ 6,1; 9,5]	0,008
Гликированный гемоглобин, %	6,2 [ 5,8; 6,7]	6,2 [ 5,8; 6,7]	6,4 [ 6,0; 7,8]	0,078
АСТ, Ед/л	33 [ 22; 45]	31 [ 21; 42]	46 [ 29; 62]	0,002
АЛТ, Ед/л	28 [ 18; 42]	28 [ 18; 40]	27 [ 21; 51]	0,205
ЛДГ, Ед/л	293 [ 235; 390]	282 [ 230; 360]	450 [ 313; 601]	0,001
IL-6, пг/мл	14,7 [ 5,36; 45,1]	12,1 [ 4,7; 37,2]	45,4 [ 14,1; 78,5]	0,002
Креатинин, мкмоль/л	79 [ 67; 95]	79 [ 66; 93]	80 [ 68; 95]	0,670
СКФ, мл/мин/1,73 м²	84 [ 63; 97]	86 [ 65; 98]	74 [ 56; 92]	0,125
Лейкоциты	6,5 [ 4,7; 8,5]	6,2 [ 4,6; 8,2]	8,1 [ 5,9; 10,1]	0,005
Нейтрофилы ×10⁹	4,6 [ 3,0, 6,7]	4,2 [ 2,9; 6,2]	6,5 [ 4,2; 8,5]	0,002
Лимфоциты ×10⁹	1,1 [ 0,7; 1,4]	1,1 [ 0,7; 1,4]	1,1 [ 0,8; 1,4]	0,921
Гемоглобин, г/л	135 [ 125; 147]	135 [ 126; 147]	136 [ 122; 148]	0,876
Гематокрит, %	41 [ 37; 44]	41 [ 37; 44]	40 [ 37; 43]	0,816
Тромбоциты ×10⁹	202 [ 162; 262]	202 [ 162; 259]	206 [ 165; 303]	0,401
СОЭ, мм/ч	35 [ 25; 50]	34 [ 25; 49]	42 [ 23; 53]	0,363
D-димер, нг/мл	264,5 [ 178,7; 514,2]	242 [ 165; 417]	420 [ 231; 1383]	0,002
Ферритин, нг/мл	452,0 [ 245,5; 908,0]	441 [ 228; 868]	803 [ 326; 1059]	0,025
СРБ, мг/л	67 [ 31; 140]	61 [ 28; 111]	170 [ 62; 232]	0,001

Тяжесть заболевания увеличивалась с возрастом и коррелировала с клиническими (ЧДД, SpO2) и лабораторными показателями — уровнем глюкозы крови и воспалительными маркерами (лейкоциты, нейтрофилы, АСТ, ЛДГ, IL-6, D-димер, СРБ, ферритин) (табл. 3, 4).

**Table:** Таблица 3. Корреляция клинико-лабораторных параметров со степенью тяжести инфекции * коэффициент ранговой корреляции Спирмена.Примечания: ЧДД — частота дыхательных движений, SpO2 — парциальное давление кислорода, АСТ — аспартатаминотрансфераза, ЛДГ — лактатдегидрогеназа, IL-6 — интерлейкин 6, СРБ — С-реактивный белок.

Параметр	r*	р
Возраст, лет	0,17	0,026
ЧДД в минуту	0,33	<0,001
SpO2, %	-0,39	<0,001
Глюкоза, ммоль/л	0,20	0,008
Лейкоциты	0,21	0,004
Нейтрофилы	0,24	0,002
АСТ, Ед/л	0,24	0,002
ЛДГ, Ед/л	0,33	<0,001
IL-6, пг/мл	0,26	0,002
D-димер, нг/мл	0,24	0,002
СРБ, мг/л	0,32	<0,001
Ферритин, нг/мл	0,17	0,025

**Table:** Таблица 4. Оценка влияния клинико-лабораторных данных на тяжесть инфекции,определенная методом бинарной логистической регрессии Примечания: ЧДД — частота дыхательных движений, SpO2 — парциальное давление кислорода, АСТ — аспартатаминотрансфераза, ЛДГ — лактатдегидрогеназа, IL-6 — интерлейкин 6, СРБ — С-реактивный белок.

Параметр	OШ (95% ДИ)	p
ЧДД в минуту	1,07 (1,03–1,18)	0,002
SpO2, %	0,89 (0,83–0,96)	0,004
Глюкоза, ммоль/л	1,14 (1,01–1,30)	0,034
Лейкоциты	0,71 (0,43–1,14)	0,185
Нейтрофилы	1,66 (0,95–2,90)	0,074
АСТ, Ед/л	0,99 (0,97–1,00)	0,281
ЛДГ, Ед/л	1,00 (1,00–1,01)	0,001
ИЛ-6, пг/мл	1,00 (0,99–1,00)	0,330
D-димер, нг/мл	1,00 (1,00–1,00)	0,325
СРБ, мг/л	1,00 (1,00–1,01)	0,001
Ферритин, нг/мл	1,00 (1,00–1,00)	0,342

## Анализ влияния компонентов РАС, полиморфизма гена ACE2 и терапии блокаторами РАС на тяжесть проявлений COVID-19.

Предшествующая развитию COVID-19 терапия блокаторами РАС включала иАПФ (эналаприл, периндоприл, лизиноприл, рамиприл) — у 35 пациентов (20%) и БРА (лозартан, валсартан, телмисартан, кандесартан) — у 15 пациентов (8,6%), кроме того, пациенты получали бета-блокаторы — 37 (21%), блокаторы кальциевых каналов — 8 (4,6%), диуретики — 19 (11%), статины — 12 (6,9%), антитромбоцитарную терапию — 22 (12,7%) пациента.

Между группами среднетяжелого и тяжелого течения COVID-19 не было выявлено статистически значимых различий в соотношении пациентов, получающих блокаторы РАС: χ²=0,208, р=0,648 для иАПФ и χ²=1,15, р=0,283 для БРА соответственно. Терапия блокаторами РАС не оказывала влияния на тяжесть течения коронавирусной инфекции по показателю ОШ (рис. 1).

**Figure:**
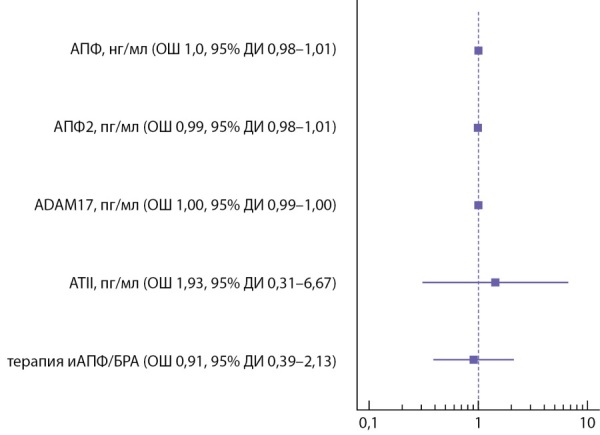
Рисунок 1. Оценка влияния уровня компонентов РАС в крови и предшествующей терапии иАПФ/БРА до развития инфекции на тяжесть течения COVID-19 (ОШ, 95% ДИ).

При сравнении пациентов по уровню компонентов РАС не было выявлено статистически значимых различий как в целом между группами среднетяжелого и тяжелого течения COVID-19, так и при гендерном распределении показателей компонентов РАС (табл. 5, 6).

**Table:** Таблица 5. Уровни исследуемых компонентов РАС у пациентов среднетяжелого и тяжелого течения COVID-19 * Критерий Манна–Уитни (U-тест).Примечания: АПФ — ангиотензинпревращающий фермент, ADAM17 — дезинтегрин и металлопротеиназа, АПФ2 — ангиотензинпревращающий фермент 2 типа.

Параметр	Общая (n=173) Ме [ Q1; Q3]	Среднее (n=144) Ме [ Q1; Q3]	Тяжелое (n=29) Ме [ Q1; Q3]	р*
АПФ, нг/мл	154,6 [ 110,7; 211,1]	158,4 [ 109,7; 214,6]	143,9 [ 111,5; 195,4]	0,544
Ангиотензин II, пг/мл	0,16 [ 0,11; 0,29]	0,15 [ 0,11; 0,26]	0,20 [ 0,10; 0,37]	0,054
ADAM17, пг/мл	101,8 [ 67,2; 185,0]	101,8 [ 67,2; 186,6]	103,4 [ 66,9; 166,2]	0,836
АПФ2, пг/мл	26,7 [ 13,6; 94,5]	27,4 [ 13,0; 94,6]	19,7 [ 14,3; 94,7]	1,0

**Table:** Таблица 6. Уровни исследуемых компонентов РАС у пациентов среднетяжелого и тяжелого течения COVID-19 в зависимости от пола (муж./жен.) * Критерий Манна–Уитни (U-тест).Примечания: АПФ — ангиотензинпревращающий фермент, ADAM17 — дезинтегрин и металлопротеиназа, АПФ2 — ангиотензинпревращающий фермент 2 типа.

Параметр	Мужчины	Женщины
среднее (n=79) Ме [ Q1; Q3]	тяжелое (n=13) Ме [ Q1; Q3]	среднее (n=65) Ме [ Q1; Q3]	тяжелое (n=16) Ме [ Q1; Q3]
АПФ, нг/мл	143,7 [ 103,1; 189,9]	147,9 [ 101,9; 197,7]	155,2 [ 113,1; 242,7]	147,9 [ 101,9; 197,7]
р= 0,695	р=0,873
Ангиотензин II, пг/мл	0,14 [ 0,10; 0,30]	0,14 [ 0,08; 0,21]	0,10 [ 0,12; 0,25]	0,22 [ 0,11; 0,61]
р=0,726	р=0,196
ADAM17, пг/мл	124,2 [ 62,6; 184,2]	91,2 [ 71,2; 238,8]	108,4 [ 67,2; 210,2]	91,2 [ 57,6; 133,3]
р=0,824	р=0,150
АПФ2, пг/мл	25,6 [ 6,7; 38,7]	54,7 [ 13,0; 153,0]	32,5 [ 17,0; 95,3]	33,6 [ 17,4; 77,0]
р=0,512	р=0,937

На основании полученных данных был выполнен регрессионный анализ с оценкой вероятности варианта течения инфекции в зависимости от исследованных факторов по отношению шансов (ОШ): уровень компонентов РАС не влиял на тяжесть течения коронавирусной инфекции (рис. 1).

Распределение генотипов rs2106809 полиморфизма гена ACE2 в соответствии со степенью тяжести инфекции представлено в таблице 7. Полиморфизм rs2106809 у женщин и мужчин проанализирован отдельно, поскольку ген ACE2 расположен на Х-хромосоме. Установлено, что распределение аллелей и генотипов полиморфизма rs2106809 гена ACE2 не различалось между группами в зависимости от тяжести заболевания для обоих полов (χ²=1,35, р=0,071 у мужчин, χ²=5,28, р=0,244 у женщин; табл. 7).

**Table:** Таблица 7. Распределение генотипов полиморфизма rs2106809 гена ACE2 в соответствии со степенью тяжести COVID-19

SNP	Пол	Генотип	Течение заболевания	χ²	р
Среднее, n (%)	Тяжелое, n (%)
rs2106809	женский	АА	50 (76,9)	8 (50)	5,28	0,071
АG	13 (20)	6 (37,5)
GG	2 (3)	2 (12,5)
мужской	АА	62 (78,5)	12 (92,3)	1,35	0,244
GG	17 (21,5)	1 (7,6)
AG	-	-

## ОБСУЖДЕНИЕ

Степень экспрессии АПФ2 в различных клетках и тканях, опосредованная возрастом, хроническими заболеваниями, фармакологической терапией может быть критическим фактором, определяющим восприимчивость организма к инфицированию и предрасположенность к более тяжелому течению заболевания. Результаты эпидемиологических исследований и метаанализов свидетельствуют о риске более тяжелого течения COVID-19 у мужчин старше 60 лет с хроническими заболеваниями, преимущественно артериальной гипертензией (21,1%), сахарным диабетом (9,7%) и кардиоваскулярной патологией (8,4%) [11]. Наше исследование также подтверждает предыдущие сообщения о наличии независимой связи возраста с тяжестью течения COVID-19. Однако достоверных гендерных различий между группами среднетяжелого и тяжелого течения заболевания и увеличения частоты коморбидных состояний в группе тяжелого течения заболевания выявлено не было. Более высокая степень экспрессии АПФ2 у молодых людей (количество рецепторов АПФ2 с возрастом снижается во всех тканях), у женщин по сравнению с мужчинами несколько противоречит сложившемуся «паттерну» о преимущественно тяжелом течении заболевания у пожилых мужчин и заставляет предположить, что уровень экспрессии АПФ2 не является ключевым фактором, определяющим прогноз пациентов с COVID-19. Одним из объяснений этого парадокса могут быть посттрансляционные изменения, регулирующие уровень белка и баланс его мембраносвязанной и растворимой форм [2]. Показано, что АПФ2 может подвергаться ADAM17 опосредованному «отщеплению» от эндотелиальных клеток, что приводит к высвобождению эктодомена с каталитической и биологической активностью в циркуляцию, усиливающему провоспалительный ответ [12]. Таким образом, более тяжелое течение коронавирусной инфекции у пожилых пациентов с хроническими заболеваниями может быть обусловлено изменением активности АПФ2 и его рецепторов, еще более усугубляющим имеющиеся негативные патофизиологические изменения.

Плохой гликемический контроль и ожирение также являются серьезными факторами риска тяжелого течения заболевания, положительно коррелируя с госпитализацией и потребностью в механической вентиляции легких [13]. По данным нашего исследования, у пациентов в группе тяжелого течения заболевания отмечена тенденция к более высоким значениям ИМТ и показателям гликемии. Отсутствие статистически значимых различий в уровне ИМТ и гликированного гемоглобина, вероятно, обусловлено небольшим размером выборки и отсутствием контрольной группы с легким течением заболевания.

В нашем исследовании не выявлено достоверных различий в уровнях именно циркулирующих компонентов РАС (АПФ, АПФ2, ангиотензина II, ADAM17) в зависимости от степени тяжести коронавирусной инфекции. Однако необходимо учитывать различия не только в сывороточном уровне АПФ2, но и тканевом, включая эндотелий сосудов, внутрипочечные сосуды, почечный тубулярный эпителий, который, безусловно, также может значимо различаться у пациентов, непосредственно влияя на уровень АТ (1–7) в тканях [14].

Ген ACE2 находится на Х-хромосоме. Для большинства генов, располагающихся на Х-хромосоме, разница в количестве копий Х между женским ХХ и мужским XY набором хромосом уравновешивается дозовой компенсацией генов с формированием транскрипционно молчащей одной из двух Х-хромосом у женщин на ранних стадиях эмбрионального развития. Однако показано, что ген ACE2 относится к классу «убегающих генов», поскольку. расположен в Xp22.2-области, где гены могут избежать Х-инактивации. Более того, хромосомная инактивация гена ACE2 имеет гетерогенный тканезависимый характер, что может приводить к фенотипическому разнообразию и различиям между полами [15]. У женщин рецептор, связывающий домен SARS-CoV-2, может распознавать АПФ2, экспрессируемый любой из двух хромосом, но не может идеально связываться с теми же последовательностями, экспрессируемыми на второй хромосоме, что теоретически позволяет несвязанному АПФ2 катализировать расщепление ангиотензина II до АТ (1–7), уменьшая таким образом неконтролируемое повреждение легких. Функциональные полиморфизмы, определяющие экспрессию гена ACE2, также могут быть ассоциированы с большим количеством мембрано-сцепленных вирусных связывающих участков, повышающих предрасположенность к инфекции. Таким образом, риск тяжелого течения может повышаться у мужчин, являющихся носителями неблагоприятных полиморфных вариантов, ввиду наличия только одной копии Х-сцепленного гена ACE2. Также имеются предположения, что rs2106809 полиморфизм гена ACE2 может приводить к снижению сывороточного уровня АПФ2, что, учитывая двойной эффект в отношении гипертонии и противовирусной активности, может частично объяснять частоту гипертонии (58%) у тяжелобольных пациентов с COVID-19 [16]. Результаты нашего исследования не выявили гендерных различий и взаимосвязи между распределением rs2106809 полиморфизма гена ACE2 и тяжестью коронавирусной инфекции. Эта гипотеза, а также связь других полиморфизмов rs2285666 гена ACE2 и I/D гена ACE также не была подтверждена в работе S. Karakas и соавт. [17].

Существуют различные гипотезы о возможных патофизиологических механизмах влияния терапии иАПФ/БРА на течение коронавирусной инфекции. Согласно одной из них, ингибирование АПФ приводит к снижению уровня АТ I, что по механизму отрицательной обратной связи в конечном итоге ведет к увеличению количества рецепторов АПФ2 и, как следствие, увеличению мест связывания SARS-CoV-2, повышая риск инфицирования. Однако результаты исследования Р. Zhang и соавт. показали, что смертность среди госпитализированных пациентов с гипертонией, получавших терапию ИАПФ/БРА, составила 3,7% по сравнению с 9,8% пациентов с гипертонией без соответствующей терапии [18]. Ряд исследователей полагают, что применение ингибиторов РАС, напротив, может приводить к повышению уровня растворимого АПФ2, способного оказывать нейтрализующее действие при связывании с Fc-фрагментом антител, тем самым ограничивая инфекцию SARS-Cov-2 [19]. По данным нашего исследования не выявлено ассоциации и увеличения риска тяжелого течения заболевания у пациентов, получавших антигипертензивную терапию блокаторами РАС. Согласно данным Всероссийского ретроспективного исследования пациентов с СД и COVID-19, прием антигипертензивной терапии иАПФ/БРА на доинфекционном этапе не влиял на тяжесть течения заболевания, более того, выявлена тенденция к снижению риска летального исхода [20].

Ряд клинических сообществ, включая Американскую кардиологическую ассоциацию (АНА), Американский кардиологический колледж (АСС), Американское общество по сердечной недостаточности (HFSA) и Европейское общество кардиологов и артериальной гипертензии (ESC/ESH), настоятельно не рекомендуют отменять терапию блокаторами РАС (иАПФ/БРА) при COVID-19 при отсутствии четких клинических доказательств их вреда.

## Ограничения исследования

Одними из основных ограничений нашего исследования являются поперечный дизайн со сплошным набором пациентов, отсутствие контрольной группы и небольшой размер выборки, особенно в группе тяжелого течения COVID-19, что могло повлиять на отсутствие условий для выявления статистически значимых различий в уровне компонентов РАС и распределении генотипов полиморфизма rs2106809 гена ACE2, о чем свидетельствует широкий ДИ для ряда изученных компонентов. Отсутствие влияния предшествующей терапии блокаторами РАС могло быть также обусловлено небольшим процентом пациентов, получавших ее до развития заболевания. Также в работе не оценивался уровень АТ 1–7 — компонента контррегуляторной оси, дисбаланс синтеза которого мог влиять на тяжесть течения заболевания.

## Направления дальнейших исследований

Результаты нашего исследования могут направить текущие исследования в области изучения генетических особенностей течения коронавирусной инфекции для определения других генов-кандидатов, полиморфных вариантов в более крупных исследованиях. Также необходимо учитывать, что, несмотря на минимальное функциональное влияние отдельных полиморфизмов, они могут находиться в тесном взаимодействии с рядом других полиморфизмов, формирующих гаплотип, непосредственно влияющий на экспрессию гена. Кроме того, появляется все больше данных о влиянии эпигенетических механизмов, таких как метилирование ДНК, гистоновые модификации, на экспрессию генов. Дальнейший поиск генетических закономерностей в формировании иммунного ответа, определяющего особенности клинического течения заболевания, позволит стратифицировать людей по степени риска и определять приоритетность защиты тех, кто нуждается в превентивной терапии.

## ЗАКЛЮЧЕНИЕ

Индивидуальные различия в клинических проявлениях и исходах коронавирусной инфекции могут быть обусловлены как особенностями врожденного или адаптивного иммунитета, так и эпигенетическими изменениями экспрессии гена рецептора ACE2, полиморфизмами других генов, оказывающих влияние на патогенез COVID-19 (провоспалительные цитокины, факторы коагуляции и т.д.). В нашем исследовании гипотеза о влиянии активации компонентов РАС (АПФ, АПФ2, АТ II, ADAM17) и полиморфизма гена ACE2 на тяжесть течения COVID-19 не подтвердилась. Степень тяжести клинических проявлений COVID-19 значимо коррелировала с уровнем стандартных воспалительных маркеров, что указывает на общие принципы течения инфекции как системного воспаления вне зависимости от генетического и функционального статуса РАС.

Негенетические детерминанты, определяющие гендерные различия в тяжести течения заболевания, могут включать как различия в частоте курения, так и особенности регуляции иммунной системы половыми гормонами или половые различия в регуляции РАС. Взаимосвязь между полом и прогнозом заболевания также требует верификации. Таким образом, перспективным представляется дальнейшее изучение совместного влияния различных полиморфных вариантов генов компонентов РАС и других систем, которые могут определять гендерные и этнические особенности течения заболевания.

## ДОПОЛНИТЕЛЬНАЯ ИНФОРМАЦИЯ

Источники финансирования. Сравнительный анализ стандартных лабораторных маркеров тяжести течения COVID-19 выполнен в рамках исследования по государственному заданию Минздрава России 122012100183-1.

Конфликт интересов. Авторы декларируют отсутствие явных и потенциальных конфликтов интересов, связанных с содержанием настоящей статьи. Компания ООО «КРКА ФАРМА» не принимала участия в сборе, анализе и интерпретации полученных данных.

Участие авторов. Все авторы одобрили финальную версию статьи перед публикацией, выразили согласие нести ответственность за все аспекты работы, подразумевающую надлежащее изучение и решение вопросов, связанных с точностью или добросовестностью любой части работы.

Благодарности. Авторы выражают благодарность компании ООО «КРКА ФАРМА» за помощь в реализации исследования.
